# Effectiveness and Toxicities of Intensity-Modulated Radiation Therapy for Patients with T4 Nasopharyngeal Carcinoma

**DOI:** 10.1371/journal.pone.0091362

**Published:** 2014-03-07

**Authors:** Fang-fang Kong, Hongmei Ying, Cheng-run Du, Shuang Huang, Jun-jun Zhou, Chao-su Hu

**Affiliations:** 1 Department of Radiation Oncology, Fudan University Shanghai Cancer Center, Shanghai, P.R. China; 2 Department of Oncology, Shanghai Medical College, Fudan University, Shanghai, P.R. China; Van Andel Institute, United States of America

## Abstract

**Objective:**

To evaluate the effectiveness and toxicities in T4 nasopharyngeal carcinoma (NPC) using intensity-modulated radiotherapy (IMRT) combined with chemotherapy.

**Methods:**

This is a retrospective analysis of 81 patients treated with intensity-modulated radiotherapy (IMRT). All the primary tumors were attributed to T4 stage according to the AJCC2010 staging system. And the distribution of disease by N stage was N0 in 13.6%, N1 in 30.9%, N2 in 37%, and N3 in 18.5%. Cisplatin-based chemotherapy was offered to all patients. Radiotherapy-related toxicities were graded according to the Acute and the Late Radiation Morbidity Scoring Criteria of the Radiation Therapy Oncology Group (RTOG) scoring criteria. Chemotherapy-related toxicities were graded by National Cancer Institute Common Toxicity Criteria (NCI-CTC) version 3.0. Prognostic factors were assessed by univariate analysis.

**Results:**

With a median follow-up of 37 months, 12 patients experienced local regional failure and total distant metastasis occurred in 18 patients, representing the major mode of failure. Ten patients died. Among them, 70% died of distant metastasis. The 3-year actuarial rates of local failure–free survival (LFFS), regional failure–free survival (RFFS), distant failure–free survival (DFFS), overall survival (OS), and progression–free survival (PFS) were 83.8%, 97.4%, 81.3%, 90%, and 69.7%, respectively. Acute and late toxicities were mild or moderate.

**Conclusions:**

IMRT provides excellent local-regional control for T4 NPC. Distant metastasis remains the major cause of treatment failure. Further explorations of the sequence and regimen of systemic therapy are needed in the future.

## Introduction

According to the AJCC 2010 staging system, T4 nasopharyngeal carcinomas (NPCs) are characterized by tumor with intracranial extension and/or involvement of cranial nerves, hypopharynx, orbit, or with extension to the infratemporal fossa/masticator space. Radiotherapy (RT) of T4 NPC is technically challenging. Since the location of the tumor has a close contact with the base of skull, the brainstem, spinal cord, optical nerve and optic chiasma, radiation is hindered by dose limitations on these organs at risk. Prescribing an excessive dose can result in significant complications, lowering patients’ quality of life [1]. Therefore, the local control of T4 NPC is poor. After conventional two-dimensional RT, the local recurrence rate ranges from 30% to 60% [2]–[5].

Intensity-modulated radiotherapy (IMRT) is an ideal radiation modality for NPC, due to its capability of delivering high radiation dose to the target while sparing the adjacent organs [6]–[9]. Encouraging results of NPC treated with IMRT have been reported. Kwong[10] and Su[11] reported a 3- and 5- year local control rate of 100% and 97.7% for early-stage NPC treated with IMRT alone. For locoregionally advanced NPC, 2- and 5-year local control rate of 82–93% and 94.9% can be achieved after effective chemotherapy combine with IMRT [12]–[14]. Although the effectiveness of IMRT for NPC has been confirmed by number of studies, the data analyzing clinical outcomes about T4 NPC are relatively rare. Therefore, in this study, we retrospectively reviewed our 3-year treatment results for patients with T4 NPC treated with IMRT, aiming to evaluate long-term effectiveness and toxicities of IMRT in T4 NPC patients.

## Materials and Methods

### Patients and Pretreatment Evaluations

From September 2007 to April 2012, 81 pathologically diagnosed T4 NPC patients without distant metastases treated by definitive IMRT in Shanghai Cancer Center of Fudan University were enrolled in this study. The pretreatment workup included a complete history and physical examination, complete blood counts, blood chemistries, endoscopy, magnetic resonance imaging (MRI) of the nasopharynx and neck, chest computed tomography (CT) or radiography, abdominal ultrasound, and emission computed tomography (ECT). All patients underwent disease restaging according to the AJCC 2010 staging system. This study was approved by the Institutional Review Boards of the Shanghai Cancer Center of Fudan University. All the patients provide their written informed consent to participate in this study. For children enrolled in this study, written informed consent was obtained from their guardians.

### Intensity-modulate Radiotherapy

#### Immobilization and simulation

Patients were immobilized in the supine position with a thermoplastic head and shoulder mask. Intravenous contrast-enhanced CT using slice thickness of 5 mm was performed for planning. Image fusion of the T1 sequences with gadolinium enhanced MRI was performed with the CT simulation images for target delineation. The CT data were imported to treatment planning system (TPS) for treatment design.

#### Target delineation

The target volumes were defined in accordance with the International Commission on Radiation Units and Measurements Reports 50 and 62. The primary gross tumor volume (GTV_P) and involved lymph nodes (GTV_N) included all gross tumors was determined by imaging, clinical, and endoscopic findings. The enlarged retropharyngeal nodes were outlined together with primary GTV, as the GTV_P on the IMRT plans.

Two clinical target volumes (CTVs) were defined in our radiotherapy: CTV1 and CTV2. The CTV1 was defined as the high-risk region that included GTV_P plus 5- to 10-mm margin; CTV1 should also encompass the entire nasopharynx, skull base, parapharyngeal space, retropharyngeal lymph nodal regions, clivus, inferior sphenoid sinus, pterygoid fossae, the posterior third of the nasal cavity and maxillary sinuses, and any high risk nodal regions, including the bilateral upper deep jugular nodes, and the near station of the positive lymph nodes. CTV2 was defined as lymph nodal regions at low risk including the lymph nodal regions of the neck which were not encompassed in the CTV1. The PTV_C would encompass the CTV with a 3-mm margin in all directions. However, when the CTV was near critical organs, such as the brainstem, spinal cord, PTV_C was generated as small as 1 mm.

The organs at risk (OAR) include the spinal cord, brain stem, optic chiasm, optic nerves, eyeballs, lens, temporal lobes, parotid glands, oral mucosa, larynx and temporomandibular joints. A 5-mm margin was added to the spinal cord and brainstem during optimization to form the planning organ-at-risk volume (PRV).

#### Treatment planning and delivery

All patients were treated with external-beam radiation therapy using 6-MV photons, 7–9 radiation fields. The treatment technique was simultaneous integrated boost (SIB) technique. The prescribed dose was 70.4 Gy to PTV_G (GTV +5 mm) and 66 Gy to PTV_N (GTV_N +5 mm) in 32 fractions. The dose delivered to PTV_C (CTV +3 mm) for subclinical disease and regional lymphatics was 60 Gy at high risk and 54 Gy at low risk in 32 fractions. All patients were treated one fraction per day, 5 days per week. The volume of PTV received less than 95% of the prescription dose should not exceed 1%. There should not be more than 110% of the prescription dose in or out of the PTV. The dose received by each organ at risk was limited to tolerance according to the RTOG 0225 protocol. The dose distribution was also examined slice by slice on the CT images.

### Chemotherapy

Chemotherapy including neoadjuvant chemotherapy, concurrent chemotherapy and adjuvant chemotherapy were given to all patients. The most common regimen of neoadjuvant and adjuvant chemotherapy included two to three cycles of TP (docetaxel 60 mg/m^2^/day, day 1, cisplatin 25 mg/m^2^/day, days 1–3), TPF (docetaxel 60 mg/m^2^/day, day 1, cisplatin 25 mg/m^2^/day, days 1–3, and 5-fluorouracil 0.5 g/m^2^/day, days 1–3), or GP (gemcitabine 1 g/m^2^/day, day1, day8, cisplatin 25 mg/m^2^/day, days 1–3) regimen. Induction chemotherapy was given every 3 weeks. Four weeks after the completion of RT, the adjuvant chemotherapy was administered every 3 weeks. Concurrent chemotherapy consisted of 80 mg/m^2^ of cisplatin every 3 weeks for 2 to 3 cycles.

### Patient Evaluation

All patients were evaluated weekly for treatment response and toxicities during radiation therapy. After IMRT, patients were clinically evaluated at predefined intervals, typically every 3 months in the first 2 years, every 6 months from the third year to the fifth year, and annually thereafter. Each follow-up included examination for the nasopharynx and palpation of neck nodes. MRI of the nasopharynx, chest CT scan, and ultrasound of abdomen were performed 3 months after the completion of IMRT and every 6–12 months thereafter. For patients with N3 disease, emission computed tomography (ECT) was routinely done annually after the completion of IMRT. Additional tests were ordered when indicated to evaluate local or distant relapse.

Radiotherapy-related toxicities were graded according to the Acute and the Late Radiation Morbidity Scoring Criteria of RTOG. Chemotherapy-related toxicities were graded by National Cancer Institute Common Toxicity Criteria (NCI-CTC) version 3.0.

### Statistical Analysis

The follow-up time was calculated from the day of the first treatment. The Statistical Package for Social Sciences (SPSS version 16.0) software was used for statistical analysis. The Kaplan-Meier method was used to estimate the cumulative local failure–free survival (LFFS), regional failure–free survival (RFFS), distant failure–free survival (DFFS), overall survival (OS), and progression–free survival (PFS). Univariate analysis was performed using log-rank test.

## Results

### Patient Characteristics

Among the 81 patients, there were 59 males and 22 females. WHO type II and III were in 20 and 61 patients, respectively. The N stages according to MRI were N0 disease for 11 patients (13.6%), N1 disease for 25 patients (30.9%), N2 disease for 30 patients (37%) and N3 disease for 15 patients (18.5%). Overall stages were Stage IVA for 66 patients (81.5%) and Stage IVB for 15 patients (18.5%). All patients received chemotherapy. The patient characteristics and treatment details are summarized in Table 1.

**Table 1:** Patient characteristics.

Characteristic	Patients (%)
Gender	
Male	59 (72.8)
Female	22 (27.2)
Age (yr)	
Median	47
Range	9–75
WHO histologic type	
II	20 (24.7)
III	61 (75.3)
Node classification	
N0	11 (13.6)
N1	25 (30.9)
N2	30 (37)
N3a	1 (1.2)
N3b	14(17.3)
Stage	
IVA	66(81.5)
IVB	15(18.5)
IMRT treatment duration(days)	
Median (range)	45(40–55)

WHO World Health Organization.

### Local Control and Survival

**Figure 1:**
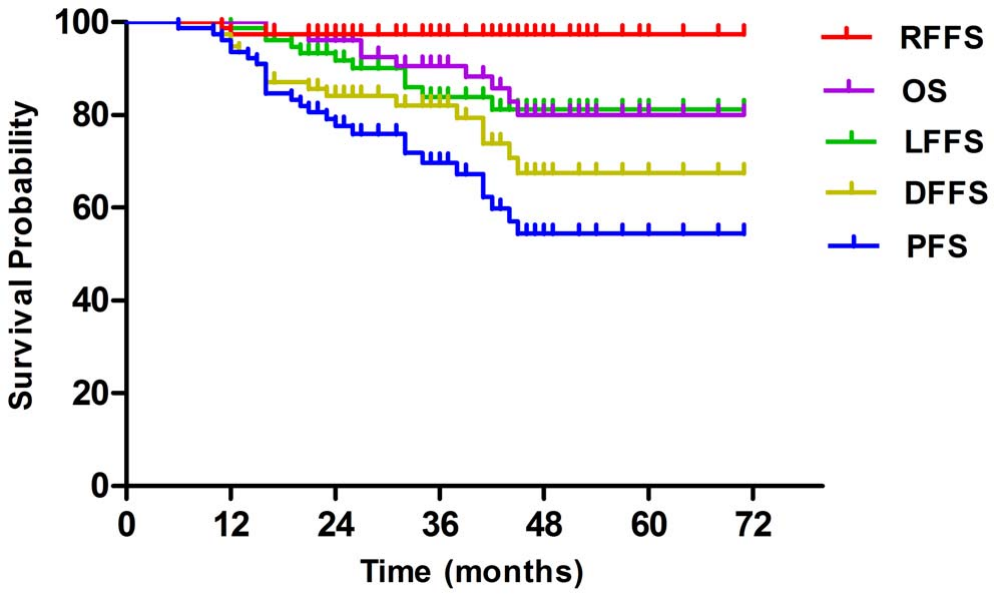
Kaplan-Meier curves showing local failure-free survival (LFFS), regional failure-free survival (RFFS), distant failure-free survival (DFFS), overall survival (OS), and progression-free survival (PFS) of patients.

**Figure 2:**
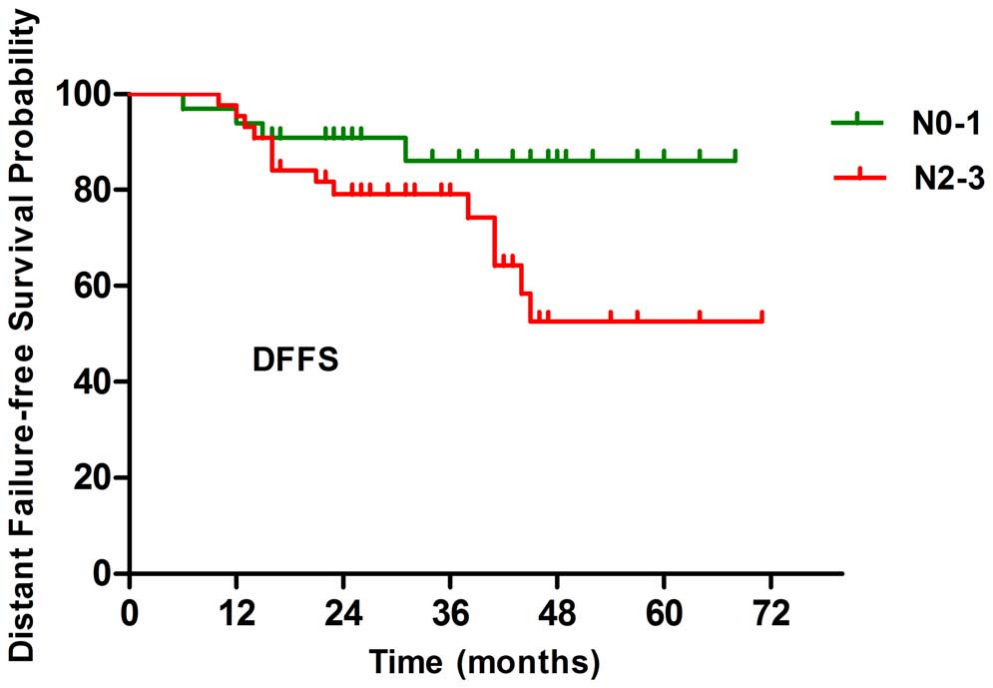
Kaplan-Meier curves showing distant failure-free survival (DFFS) between N0–1 and N2–3 patients.

The median follow-up time was 37 months, with a range from 3 months to 71 months. The 3-year LFFS, RFFS, DFFS, OS and PFS were 83.8%, 97.4%, 81.3%, 90% and 69.7%, respectively (Figure 1). The overall 3-year DFFS for patients with Stage N0–1 and N2–3 was 86.1% and 79.1% (*P* = 0.047) (Table 2, Figure 2). Ten patients died. Among them, 7 (70%) and 2 (20%) patients died because of distant metastasis and local recurrence, respectively. The remaining 1 patient died of unknown causes (10%).

**Table 2:** Impact of prognostic factors on treatment results by univariate analysis.

Items	3y-LFFS		3y-RFFS		3y-DFFS		3y-OS		3y-PFS	
	%	P	%	P	%	P	%	P	%	P
Gender										
Male	87.3	0.190	96.4	0.396	77.8	0.719	80.8	0.582	71.1	0.531
Female	74.4		100		90.5		92.9		66.0	
Age(yr)										
<60	83.2	0.533	96.9	0.559	79.7	0.606	88.1	0.175	68.4	0.520
≥60	88.9		100		90.9		100		79.5	
N stage										
N0–1	85.8	0.934	100	0.225	86.1	0.047	95.7	0.120	77.2	0.070
N2–3	82.0		95.5		79.1		86.7		63.8	

LFFS, local failure-free survival; RFFS, regional failure-free survival; DFFS, distant failure-free survival; OS, overall survival; and PFS, progression failure-free survival.

Overall disease failures (at any site) were found in 27 patients, including 9 patients who had local recurrence alone, 15 patients who had distant metastases alone, and 3 patients who had both locoregional and distant failures. Details were shown in Table 3.

**Table 3:** Incidence and site of treatment failure.

Site	Patients (n = 81)	
	No.	%
Local and/or regional		
Local only	9	
Local and distant	1	
Regional and distant	1	
Local, regional and distant	1	
Distant only	15	
Bone only	5	
Lung only	6	
Liver only	1	
Brain only	1	
Multiple location^a^	5	
Total locoregional failure	12	14.8
Total distant failure	18	22.2

a Either combinations of bone, liver brain and lung.

### Prognostic Factors

The value of various potential prognostic factors including gender, age, and N stage on predicting LFFS, RFFS, DFFS, OS and PFS were evaluated. Univariate analysis showed that N stage was a significant prognostic factor for DFFS (*P* = 0.047), favoring those with N0–1 disease. No significant prognostic factors were found to LFFS, RFFS, OS and PFS. Details were shown in Table 2.

### Toxicity

Acute and late toxicities by site and grade were detailed in Table 4. The most common radiation-related acute toxicities were dermatitis (82.7% grade 1), mucositis (51.9% grade 2) and xerostomia (71.6% grade 2). The worst acute mucositis was grade 3 in 18.5% of the patients, and no patient had grade 4 toxicity. Chemotherapy-related hematologic toxicities were also listed in Table 4. Grade 3 anemia, leukopenia, and thrombocytopenia occurred in 2.5%, 17.3% and 6.2% of the patients, respectively. 13.6% of the patients experienced grade 4 leukopenia.

**Table 4:** The frequency of acute and late toxicities for the patients.

Toxicities	No. of patients by toxicity grade (%)				
	Grade 0	Grade 1	Grade 2	Grade 3	Grade 4
The acute toxicities					
Dermatitis	0	67 (82.7)	14 (17.3)	0	0
Mucositis	0	24 (29.6)	42 (51.9)	15 (18.5)	0
Xerostomia	0	23 (28.4)	58 (71.6)	0	0
Anemia	41 (50.6)	27 (33.3)	11 (13.6)	2 (2.5)	0
Leukopenia	9 (11.1)	26 (32.1)	21 (26)	14 (17.3)	11 (13.6)
Thrombocytopenia	42 (51.9)	20 (24.7)	14 (17.3)	5 (6.2)	0
Liver dysfunction	76 (93.8)	3 (3.7)	2 (2.5)	0	0
Renal dysfunction	77 (95.1)	4 (4.9)	0	0	0
diarrhea	78 (96.3)	3 (3.7)	0	0	0
Nausea/vomiting	19 (23.5)	35 (43.2)	16 (19.8)	11 (13.6)	0
Alopecia	22 (27.2)	53 (65.4)	6 (7.4)	0	0
The late toxicities^a^					
Xerostomia	6 (7.5)	42 (52.5)	32 (40)	0	0
Hearing loss	64 (80)	7 (8.8)	5 (6.2)	4 (5)	0
Temporal lobe necrosis	74 (92.5)	5 (6.3)	1 (1.2)	0	0
Cranial nerve palsy	78 (97.5)	2 (2.5)	0	0	0
Spinal cord injury	79 (98.8)	1 (1.2)	0	0	0

a Radiotherapy-related late toxicities were defined in patients whose follow-up period was over one year.

Late toxicities were assessed in 80 patients whose follow up period was over one year. The serious late toxicities were radiation-induced temporal lobe necrosis (6 cases), hearing loss (16 cases), and cranial nerve palsy (2 cases). Xerostomia appeared to decrease with time after treatment. The number of patients with grade 2 xerostomia decreased gradually, whereas the number of patients with grade 0–1 xerostomia increased.

Six patients suffered radiation-induced temporal lobe necrosis, diagnosed by magnetic resonance imaging examination during the follow-up period. Among them, 5 patients were asymptomatic, and 1 patient suffered from mild headache and memory loss. The sites of these temporal lobe injuries were all unilateral. All of these 6 patients had primary bulky tumors with extensive skull base and intracranial tissue invasion. The average minimum, maximum and mean dose deliver to the affected side of temporal lobe were 4.7 Gy, 75.9 Gy and 28.8 Gy, respectively. The average V70 (percentage of temporal lobe volume receiving a dose of 70 Gy or more) was 7.86%.

Sixteen patients developed irreversible hearing loss. Seven of them were unilateral and 9 of them were bilateral. These injuries may be the result of the extensively use of cisplatin, and the increased dose to the inner ear, which was caused by the extensive invasion of the skull base.

For the two patients with cranial nerve palsy, one patient exhibited injury to the mandibular division of the trigeminal (V3) nerve, and the other patient presented with hypoglossal nerve (XII) and V3 injury. One patient had mild Lhermitte’s syndrome caused by radiation- induced spinal cord injury.

## Discussion

There is little controversy that IMRT has been the treatment of choice for NPC without distant metastases, especially for locoregionally advanced disease. Treatment outcomes from various centers are encouraging. Cao et al[14] published their experience of IMRT in 70 patients with T4 NPC. With a median follow-up of 26.8 months, satisfactory local control rate of 82.1% was achieved. Similar excellent 2-year clinical outcomes were reported by Ma et al[12]. Xiao et al[13] recently reported their long-term results of a phase II study of 81 locally advanced NPC patients. With a median follow-up of 54 months, only 4.9% patients experienced local recurrence. The 5-year local control rate was 94.9%. In our study, we observed a 3-year local control rate of 83.8%. This was relatively higher than that reported by Cao et al[12], lower than that reported by Xiao et al[13]. We attributed that to the higher percentage of patients received chemotherapy in our series than that in Cao et al[12] (100% *vs.* 84.3%), and the higher percentage of T4 patients in our series than that in Xiao et al[13] (100% *vs.* 40%). Considering all the above factors, the local control in our study is quite satisfactory. This further confirmed the promising role of IMRT for locally advanced NPC.

Although all patients in our study received chemotherapy, the 3-year DFFS, OS and PFS were only 81.3%, 90% and 69.7%, respectively. Distant metastasis was the major cause of death in patients after treatment. However, the DFFS and OS in the present study were relatively higher than those reported by Cao et al[14]. They reported a 2-year DFFS and OS of 73.8% and 82.5%, respectively. Except for the higher percentage of patient who received chemotherapy in this trial (100% vs. 84.3%), the sequence of chemotherapy may be another reason. Given that the compliance and tolerance of concurrent chemoradiotherapy (CCRT) for outpatients is relatively poor, most of the patients (71.6%) in our study received neoadjuvant-adjuvent chemotherapy. This was also different from that reported by Cao et al[14] in which CCRT was in the majority. The phase II clinical trial of Hui et al[15] compared concurrent cisplatin-radiotherapy with or without neoadjuvant docetaxel and cisplatin in advanced NPC have demonstrated positive OS improvement (94.1 vs. 67.7% at 3-year, P = 0.012). The phase III clinical trial of Xu et al[16] compared the efficacy, toxicities and compliance of locoregionally advanced NPC treated with neoadjuvant chemotherapy (NACT) or concurrent chemotherapy (CCRT) revealed more acute toxicities in CCRT arm and a trend of better tolerance was observed in NACT arm. Furthermore, Du et al[17] reported promising outcomes with good compliance and well-tolerated toxicities of neoadjuvant–adjuvant chemotherapy using cisplatin, fluorouracil, plus docetaxel for locoregionally advanced NPC. The 2-year estimated LR-FFS, DFFS, PFS, and OS were 96.6%, 93.3%, 89.9%, and 98.3%, respectively [17]. Therefore, for locoregionally advanced outpatients or patient who can’t tolerate CCRT, the neoadjuvant–adjuvant chemotherapy combined with IMRT could be a good alternative.

In the current study, we observed a few significant acute and late toxicities. And the toxicity profiles were acceptable and tolerable in general. The most common radiation-related acute toxicities were grade 1–2. 13.6% of the patients experienced grade 4 leukopenia. No patient needed a nasogastric tube or gastrostomy for nutritional support during the course of radiotherapy. This contrasts to a recent study by Ng et al. that reported a higher radiation-induced morbidity with IMRT in nasopharyngeal carcinoma [18]. We attributed this to the lower percentage of patients receiving concurrent chemotherapy (14.4% *vs.* 84%) in our series. The serious late toxicities were radiation-induced temporal lobe necrosis (6 cases), hearing loss (16 cases), cranial nerve palsy (2 cases), and dysphagia (1 cases). No patient had grade 4 radiation-induced toxicity.

It is worthwhile to note that several temporal lobe injuries were detected in our study, though the median follow-up time was only 37 months. These injuries may be the result of extensive invasion of the skull base and even intracranial tissue, which led to increased dose to the temporal lobes. It is reported that radiation-induced temporal lobe injuries is relate to fractional dose, the overall treatment time, and the use of chemotherapy[19]–[20]. In the present study, we found that most of the radiation hot spot was in the enhanced region of the affected temporal lobe, little was in the edema area around. It suggested that the radiation hot spot may have a close correlation to the temporal lobe injuries after IMRT. However, further explorations are still necessary. Although the damage to the temporal lobe was not extremely severe, further study is still very crucial and indispensable to achieve sufficient irradiation doses to the tumor while protecting normal organs in patients with locally advanced disease. Our study has several limitations. First, it is a retrospective study. Second, since the interval for enrollment was about 5 years, various factors such as the sequence and regimen of systemic therapy have evolved. Third, due to the relatively small sample size and short time follow-up, the current findings could only be taken as preliminary. Therefore, well-designed randomized trials and long-term follow up are needed for further research.

In conclusion, our study showed that IMRT provided excellent local-regional control for T4 NPC, with acceptable acute and late toxicities. Further research is necessary to decrease the dose given to the temporal lobes in patients with bulky tumors that extensively invade the skull base and intracranial tissue. Distant metastasis remains the major cause of treatment failure in our study. Further explorations of the sequence and regimen of systemic therapy are needed in the future.
